# Prevalence of depressive symptoms in urban primary care settings: Botswana

**DOI:** 10.4102/phcfm.v13i1.2822

**Published:** 2021-05-07

**Authors:** Keneilwe Motlhatlhedi, Keneilwe Molebatsi, Grace N. Wambua

**Affiliations:** 1 Department of Family Medicine and Public Health, Faculty of Medicine, University of Botswana, Gaborone, Botswana; 2 Department of Psychiatry, Faculty of Medicine, University of Botswana, Gaborone, Botswana; 3 Department of Psychiatry, School of Clinical Medicine, University of KwaZulu-Natal, Durban, South Africa

**Keywords:** epression, depressive symptoms, PHQ-9, prevalence, urban, primary care, Botswana, Africa

## Abstract

**Background:**

The prevalence of depression is estimated to be high in primary care settings, especially amongst people with chronic diseases. Early identification and management of depression can improve chronic disease outcomes and quality of life, however, there are many missed opportunities in primary care.

**Aim:**

This study aimed to determine the prevalence and correlates of depression and depressive symptoms in two urban primary care settings.

**Setting:**

The study was conducted at two primary care facilities in the capital city of Botswana.

**Methods:**

We administered a demographic questionnaire and the Patient Health Questionnaire-9 (PHQ-9) to adults attending two primary care facilities. The association between depressive symptoms and demographic variables was determined using Chi-square; level of significance was set at 0.05. We carried out a multivariate analysis using Kruskal-Wallis test to determine the association between demographic characteristics and depression.

**Results:**

A sample of 259 participants were recruited (66.8% women, median age 32). The mean PHQ-9 score was 8.71. A total of 39.8% of participants screened positive for depression at a cut-off of 9.0% and 35.1% at a cut-off of 10. Depressive symptoms were significantly associated with employment status and income using the Kruskal-Wallis test, χ^2^ (1) = 5.649, *p* = 0.017.

**Conclusion:**

The high rates of depressive symptoms amongst the study population highlight the need for depression screening in primary care settings. The association between unemployment and income underscore the impact of socio-economic status on mental health in this setting.

## Background

The World Health Organization (WHO) ranks depression as a leading cause of non-fatal health loss and the fourth leading cause of disability globally.^1,2^ Estimates suggest that 4% – 15% of people suffer from depression at some point in their lives, whilst the point prevalence of depression is estimated to be between 5% and 10%.^1,3,4,5^ Higher depression prevalence rates ranging from 4% to 50%, have been reported in some sub-Saharan Africa (SSA) settings.^1,4,6^ Furthermore, the prevalence of depression has been found to be higher amongst people with chronic diseases such as HIV, diabetes mellitus and cardiovascular diseases, many of whom receive care in primary care centers.^2,7,8,9,10^

The characteristic symptoms of depression include sadness, anhedonia and self-harm; the effects of the disorder extend beyond the individual and may negatively affect family life, occupational and academic functioning and the society at large.^1,9,11,12^ Although the mechanism is unclear, there is evidence that the bidirectional relationship between depressive disorders and chronic diseases may worsen physical and psychological outcome and increase the cost of healthcare.^8,11,13^ On the one hand, it is possible that the negative symptoms of depression result in poor adherence to medications and life-style modifications, which are important for improved chronic disease outcomes. On the other hand being diagnosed with a chronic disease further worsens the anxiety and negative self-assessment of a patient with depression.^8^

Early identification and management of depression may help to curb the negative impact of depression and improve the quality of life of those who suffer from the condition.^14,15^ The WHO reports that up to 85% of patients with the disorder do not receive appropriate care, many of whom are in low- and middle-income countries (LMICs).^16,17^ This insufficiency in care may be attributable to a shortage of adequately skilled mental health staff, competing health concerns and limited consultation times because of the low healthcare workers to population ratios in LMICs, particularly there is a shortage of adequately skilled mental healthcare workers.^16,18,19^ Patients with depression may initially present with somatic symptoms. Evidence indicates that depressed patients with predominantly somatic symptoms are less likely to be diagnosed with depression than those who present solely with psychological or mood symptoms.^20,21^ Most patients with depression (regardless of the predominant symptoms) are initially consulted at primary care level; primary care clinicians are therefore best suited to identify mild and moderate forms of the illness early on and to initiate appropriate management. The use of depression screening tools may enhance the ability of primary care clinicians in LMICs to identify patients with depression.

Screening for depression in primary care settings requires an understanding of the factors which predisposed patients to depression. Previous research has reported various predictors of depression, including a family history of depression or mental illness, chronic illness, substance abuse, low socio-economic status, female gender and older age.^4,12,19,22,23^ The strength of the association of these factors with depression may vary between populations, for example, low income was associated with depression in high-income countries but not in LMICs, whilst lower education status was associated with depression in some Asian countries, but not in others.^4^ In addition, even though the presence or absence of a factor may be positively associated with depression, the inverse may not necessarily be true. For example, in one South African study, unemployment and low income were positively associated with depression, but surprisingly the relationship with education was not that clear, whilst higher education attainment seemed protective as there was no association between depression and lower education attainments.^12^ These differences show the importance of understanding population-specific factors associated with depression in order to enhance screening efficiency as a first step to enhancing diagnosis and treatment of primary care patients with depression.

Previous studies in Botswana have estimated a depression prevalence of 28% – 35%.^24,25^ However, these studies targeted HIV-infected people and may not be reflective of the primary care population in Botswana. We did not find any studies reporting the prevalence and correlates of depression in the general primary care population in Botswana. This knowledge would help identify the specific sub-population of primary care patients who may benefit from depression screening and appropriate management in Botswana. The aim of this study was to determine the prevalence and correlates of depressive symptoms in two urban primary care settings in Botswana.

## Methods

This article is a sub-analysis of data from a validation study of the Patient Health Questionnaire-9 (PHQ-9) and depression screening questionnaire in Botswana.^26^ The study was conducted in two primary care outpatient health facilities in Gaborone, the capital city of Botswana. One facility, Old Naledi Clinic, is located in a low-income area whilst the other, Village Clinic, is located in a middle- to high-income area. Both are public health facilities offering free healthcare services to citizens. Data were collected for this study on a daily basis during weekdays between May 2019 and June of 2019. Trained research assistants (RAs) enrolled participants from the general outpatient departments at each site; the RAs explained the importance of the study and study procedures to the patients as they waited in the consultation queue. Adult patients aged above 18 years, who could speak Setswana or English (the two official languages), who were not physically acutely ill and did not have a current psychotic illness and who gave signed informed consent were enrolled consecutively. The sampling was carried out using convenience sampling, patients waiting in the queue were approached sequentially to ask if they would be interested in joining the study. Those who consented were then interviewed using a researcher designed demographic questionnaire and the PHQ-9. Patients who were found to be severely depressed or who had suicidal ideation were referred to the psychiatric nurse based at each facility for further assessment and management or referral.

## Measures

A *demographic questionnaire* was designed to capture the following data: age, gender, income bracket, occupation, education level, marital status, chronic diseases including HIV and psychiatric disease, substance use and family history of chronic disease including psychiatric conditions.

The *PHQ-9* is a 9-item depression screening tool that has been validated in several primary care settings in Southern Africa that can be self or provider administered.^27,28,29^ The patient is asked if they experience each of the nine symptoms in the questionnaire over the preceding two weeks. Each of the nine questions can be scored from 0 to 3, indicating increasing frequency from not experiencing the symptom at all to almost daily symptoms. A validation study conducted in Botswana reported the sensitivity and specificity of the PHQ-9 to be 72.4% and 76.3%, respectively, at a cut-off point of 9.0% and 68.6% and 79.6%, respectively, at a cut-off point of 10.^26^

## Statistical analysis

A sample size of 252 was calculated for the main objective of the study, which was to validate the PHQ-9 in this primary care population.^26^ Determining the prevalence of depression was the secondary aim of the study. Assuming the reference proportion of patients with depression is 20%, the study is adequately powered (80%) to detect a minimum 8% difference in depression prevalence, with a precision level of 0.05. Initial analysis showed that the data were not normally distributed, we report descriptive data as medians and inter-quartile ranges for continuous data and proportions for categorical data. To test for the association between depression and demographic variables we calculated Pearson’s Chi-square. The level of significance was set at 5%. Spearman’s rank correlation was used to determine the association between demographic characteristics and depression using a PHQ-9 cut-off of 10. Multivariate analysis was carried out using Kruskal-Wallis one-way analysis of variance (ANOVA) to determine the association between demographic characteristics and depression.

### Ethical considerations

Full ethical clearance was granted by the University of Botswana Institutional Review Board (UBR/RES/IRB/BIO/122) and Health Research and Development Division, Botswana Ministry of Health and Wellness (13/18/1). All participants gave written and informed consent to participate in the study. Confidentiality was maintained by use of unidentified data collection forms.

## Results

### Participant characteristics

The final study sample size was 259, which was slightly larger than the minimum calculated sample size 252, of these 66.8% were females, the median age was 32 years. A little over a third of participants identified as not being in a relationship (they were either single, divorced or widowed). Unemployment was high at 23.6% and about 13% had less than a primary education. Over half of the study population identified as having no chronic illness whilst 22.4% identified as being HIV positive. Table 1 shows the demographic characteristics of the participants.

**TABLE 1:** Demographic characteristics of study participants in two primary care settings in Gaborone, Botswana.

Variable	*N*	%
**Healthcare facility**		
ON	130	50.2
VI	129	49.8
**Gender**		
Female	173	66.8
Male	85	32.8
Missing	1	0.4
**Age**		
Median (years)	32	-
IQR	24–41	-
**Marital status**		
Single	64	24.8
In a relationship	88	34.1
Married	29	11.2
Widowed	8	3.1
Divorced	10	3.9
Cohabiting	59	22.9
Missing	1	0.04
**Employment status**		
Unemployed	61	23.6
Self-employed¶	36	13.9
Part-time¶	9	3.5
Casual labourer¶	9	3.5
Formal employment	125	48.3
Student	19	7.3
**Religion**		
Islam	4	1.5
Christianity	225	86.9
African traditional religion	9	3.5
Other	14	5.4
None	1	0.4
Missing	6	2.3
**Education**		
No school	6	2.3
Primary	30	11.6
Junior Secondary	85	32.8
Senior Secondary	74	28.6
Tertiary	64	24.7
**Diagnosis†‡**		
Diabetes	5	1.9
Stroke/CVA	6	2.3
HIV	57	22.0
Hypertension	44	17.3
Other	21	8.3
None	142	55.6
Missing	4	1.6
**Estimated average monthly income (BWP)**		
Below 600	46	17.8
600–2300	107	41.3
2400–4199	44	17.0
4200–6000	23	8.9
Above 6000	19	7.3
Not disclosed	19	7.3
**Substance use**		
Alcohol only	81	31.3
Dagga/cannabis only	1	0.4
Tobacco only	4	1.5
Others	33	12.7
None	138	53.3
Missing	2	0.8
**Relative with MI**		
Yes	59	23
No	181	70.7
Do not know	61	6.3
**You with MI**		
Yes	5	1.9
No	251	96.9
Missing	3	1.2

Note: 1 Botswana pula = 0.091 United States dollar at time of study.

ON, Old Naledi Clinic; VI, village clinic; IQR, interquartile range; HIV, human immunodeficiency virus infection; MI, mental illness; CVA, cerebrovascular accident; BWP, Botswana pula.

† , diagnoses as stated by patient;

‡ , more than one substance or other illicit drug;

§ , answers may not add up to 259 because more than one response possible for each participant;

¶ , informal employment.

### Prevalence of depression

Seven participants had missing data for some of the PHQ-9 questions. Thus, the PHQ-9 score could not be calculated for them. Using the remaining sample of 252, the prevalence of depression or depressive symptoms, which was 40.9%, with a 95% confidence interval (CI) (34.7, 47.2), using a PHQ-9 cut-off of nine. A slightly lower prevalence of 36.1%, with a 95% CI (30.2, 42.4) was seen when using a higher cut-off of 10.

### Factors associated with depression

Table 2 shows the bivariate analysis to determine the level of association between depression (PHQ-9 cut-off 10) and demographic factors of the participants as analysed using Chi-square, χ^2^ (or Fisher exact test where number of values expected in any cell was less than 5). A positive association was observed between depression and employment (grouped as unemployed, informal employment, formal employment or student) (χ^2^ = 15.139, and *p* = 0.002) and income, *p* = 0.020.

**TABLE 2:** Association of demographic variables and depression in two primary care settings in Gaborone, Botswana using a Patient Health Questionnaire-9 cut-off score of 10.

Variable	No depression (PHQ-9 of < 10), (*n* = 161)		Depression (PHQ-9 = 10 or above), (*n* = 91)		Chi-square value	*p*-value
	*N*	%	*N*	%		
**Gender**						
Female	102	40.6	68	27.1	3.77	0.152
Male	58	23.1	23	9.2	-	-
**Marital status**						
Single	41	25.2	19	11.7	9.923*	0.710
Married	15	9.2	14	8.6	-	-
Widowed	7	4.3	0	-	-	-
Divorced	6	3.7	4	2.5	-	-
Cohabiting	41	25.2	16	9.8	-	-
**Employment status**						
Unemployed	28	11.1	32	12.7	15.139	**0.002**
Informal employment	31	12.3	21	8.3	-	-
Formal employment	91	36.1	30	11.9	-	-
Student	11	4.4	8	3.2	-	-
**Religion**						
Islam	3	1.2	1	0.4	4.813*	0.269
Christianity	141	57.3	77	31.3	-	-
ATR	4	1.6	5	2.0	-	-
Other	6	2.4	8	3.3	-	-
None	1	0.4	0	-	-	-
**Education**						
No school	4	1.6	2	0.8	2.414*	0.668
Primary	17	6.7	11	4.4	-	-
Junior Secondary	48	19.0	34	13.5	-	-
Senior Secondary	51	20.2	22	8.7	-	-
Tertiary	41	8.7	22	8.7	-	-
**Diagnosis**						
One NCD	20	8.0	9	3.6	2.494	0.650
HIV only	27	10.8	15	6.0	-	-
Comorbidity†	11	4.4	11	4.4	-	-
None	90	36.1	52	20.9	-	-
Other	10	4.0	4	1.6	-	-
**Income**						
Below P600	18	7.2	28	11.2	15.323*	**0.020**
600–2300	69	27.7	32	12.9	-	-
2400–4199	31	12.4	12	4.8	-	-
4200–6000	15	6.0	8	3.2	-	-
Above 6000	13	5.2	6	2.4	-	-
Not disclosed	12	4.8	5	2.0	-	-
**Substance use**						
Alcohol	52	20.8	28	11.2	5.561*	0.198
Dagga	1	0.4	0	-	-	-
Tobacco	0	-	3	1.2	-	-
Other	19	7.6	12	4.8	-	-
None	89	35.6	46	18.4	-	-
**Relative with MI**						
Yes	34	13.7	24	9.6	6.793*	0.097
No	112	44.9	63	25.3	-	-
Don’t know	14	5.6	2	0.8	-	-
**You with MI**						
Yes	1	0.4	4	1.6	6.629*	**0.059**
No	158	63.5	86	34.5	-	-

Note: Bold numbers indicate statistically significant *p*-value.

PHQ-9, Patient Health Questionnaire 9; ATR, African traditional religion; NCD, non-communicable disease; MI, mental illness.

* , Fisher’s exact test.

† , more than one chronic disease in any combination.

## Discussion

The aim of this study was to determine the prevalence and correlates of depression in two urban primary care settings in Botswana. Our findings show that the point prevalence of depressive symptoms was much higher (40.9% ) 95% CI (34.7, 47.2) in this primary care population than current global estimates of depression and previous studies of depressive symptoms in primary care.^19,30^ Our findings are similar to other studies carried out in SSA, reporting a prevalence range of 30% – 56%.^6,12,17,23^ It is important to differentiate depressive symptoms as measured in this study from clinical depression, which requires a more robust diagnosis. However, adopting easy to use and freely available screening tools such as the PHQ-9 to screen high-risk patients for depression, may help clinicians in busy health settings identify patients who need further intervention. The findings of this study also support previous assertions that primary care clinicians in African settings should be equipped to diagnose and manage depressive symptoms and depression.^14,31,32^

Our analysis of the association between depression and participant demographics indicates that the proportion of those with depression was higher in lower income levels and in the unemployed.^12,20^ It has been suggested that the severity of depressive symptoms is inversely related to employment and income. Research shows that the occurrence of depression can also lead to alteration of economic productivity, a decrease of working abilities, social isolation, physical decline and difficulties in solving problems.^20^ Alternatively, being unemployed may negatively affect a person’s self-esteem and mood thus triggering depression.^33^ The association of depressive symptoms with unemployment and low income indicates that these factors may be considered as triggers for depression in this primary care population. Considering these patients as high risk for depression may warrant targeted screening and diagnosis. Including occupational rehabilitation and work placement in the depression treatment strategies of this primary care population may help alleviate their depressive symptoms.^33,34^ However, caution should be exercised when analysing this relationship because income and employment status are only part of the broader construct of social status and social support and may not adequately reflect this broader construct. The effects of income and or unemployment on depression may be mitigated by good social support; a study of 402 patients in outpatient tertiary institutions in Nigeria using the Schedule for Clinical Assessment of Neuropsychiatry (SCAN) found no association between depression and unemployment whilst a study from the United Kingdom found that accounting for debt diminished the effect of income on mental health, that is, being in debt was a stronger determinant of mental illness than income alone.^6,35^ Thus, clinicians must further understand the impact of income and unemployment on a patient’s life when assessing for depression.

Although comorbidity with depression and other chronic illnesses have been reported in several studies, we did not find a significant relationship between depression and chronic illnesses. Approximately, a third of the patients in this study identified as having a chronic illness, although this is reflective of primary care settings, these numbers may be too small to detect a difference in the prevalence of depression in patients with versus those without chronic illnesses. Previous studies in Botswana have reported about a quarter to a third of HIV patients in primary care settings had depression, this prevalence was not much different from what we found in this general primary care setting but are much higher than the prevalence we report for HIV-infected patients in this study.^24,25^ Our study was not adequately powered to detect differences in depression by chronic disease; we suggest an adequately powered study to investigate the relationship between depression and chronic illnesses in primary care may help to answer this question better.

We found that a history of previous mental health was not associated with depression in this study population. This finding is unsurprising because common mental illnesses such as anxiety are often undiagnosed. In addition, in this study there were only five people who reported a previous diagnosis of mental illness, this number is most likely insufficient to detect a significant difference.

### Limitations

This study was an observational study conducted in two primary care settings in an urban area, the findings of this study may thus be limited to similar outpatient facilities and not to the general population. The risk for depression in general outpatient facilities may also be different from the risk of depression in primary care settings targeting chronic disease patients. In addition, the study sample was obtained using convenience sample, which further contributed to the selection bias of the study. Patients who agreed to join the study may have been more likely to suspect they had depression than those who refused. This would bias the results such that the effect size was larger than the true state. However, the prevalence of chronic diseases and the proportion of unemployed people in this study are similar to previous reports from Botswana, this indicates that any selection bias may be minimal. We recommend future studies using randomised sampling techniques and community-based studies to determine the prevalence of depression in the general population.

## Conclusion

Our study is the first in Botswana to assess the prevalence of depression or depressive symptoms in patients in routine clinical care, highlighting that there is a need to include depression screening as part of care they receive. We recommend the use of PHQ-9 for depression screening in urban primary care populations in Botswana The association between depression and unemployment and income highlights the importance of social determinants of mental health. We recommend that in addition to the traditional cues for depression screening (low mood and anhedonia), clinicians should have a low threshold for depression screening in unemployed primary care populations similar to ours.
